# From Chromosomes to Precision Therapy: Clinical Cytogenetics and Cytogenomics in the Era of Genomic Medicine

**DOI:** 10.3390/genes17080879

**Published:** 2026-07-28

**Authors:** Jinglan Liu

**Affiliations:** Clinical Cytogenomics, Department of Pathology and Genomic Medicine, Sidney Kimmel Medical College, Thomas Jefferson University, Philadelphia, PA 19107, USA; jinglan.liu@jefferson.edu

Chromosomes form the foundation of both the structure and function of the human genome.

Indeed, the very word “genome” is a linguistic fusion of gene and chromosome, echoing their intrinsic biological relationship. Chromosomes serve not only as carriers of genetic information but also as key regulators of genome organization and function [1,2].

For decades, clinical cytogenetics has been central to the diagnosis of constitutional and neoplastic disorders. The field has continuously evolved toward higher resolution and greater diagnostic sensitivity, progressing from conventional G-banded karyotyping to fluorescence in situ hybridization (FISH), chromosomal microarray analysis (CMA), optical genome mapping (OGM), and long-read sequencing. Advances in sequencing technologies and bioinformatics have further expanded the detection of chromosomal abnormalities, allowing whole-exome and whole-genome sequencing to complement traditional cytogenetic methods [3]. Despite major advances in mutation-guided precision oncology, reproducing the transformative success of imatinib in chronic myeloid leukemia (CML) remains challenging [4]. Tumor heterogeneity, clonal evolution, and genomic plasticity frequently limit the effectiveness of targeted therapies. Because most cancers arise from multilayered, complex structural and functional genomic alterations, precision medicine must integrate chromosomal architecture with DNA sequence information. This Special Issue highlights how the macrostructural perspective of clinical cytogenetics provides an essential framework for advancing precision medicine [5].

The nine manuscripts included in the first edition of this Special Issue span the spectrum from conventional chromosome analysis to modern computational cytogenomics, reaffirming that chromosome biology remains fundamental to understanding human disease in the era of genomic medicine. Together, these complementary approaches expand and strengthen the diagnostic power of contemporary cytogenetics.

This Special Issue opens by revisiting the foundations of chromosome-based diagnostics. Ye et al. present a compelling perspective arguing that karyotype-level architecture fundamentally coordinates gene-level function, establishing cytogenetics as an indispensable framework in the post-genomic era. By integrating high-resolution sequencing data with chromosome-level organization, the authors reposition cytogenetics within a systems-level understanding of disease [6]. Blind spots remain inevitable in frontline diagnostic testing. Delikkaya et al. describe an exceptionally rare case of acute promyelocytic leukemia (APL) that was both cytogenetically cryptic and FISH-negative, requiring quantitative molecular testing to identify a concealed *PML::RARA* fusion. Their accompanying review of 34 similar cases underscores the importance of integrating molecular and conventional diagnostic approaches [7]. Similarly, Xia et al. report a pediatric acute myeloid leukemia case with an atypical *CBFB::MYH11* fusion on a supernumerary ring chromosome 16, demonstrating how comprehensive cytogenetic and molecular profiling enables accurate characterization of complex genomic abnormalities and supports successful targeted therapy [8]. A recurring theme throughout this volume is that increasingly sensitive genomic technologies should complement, rather than replace, classical cytogenetics. Liehr et al. demonstrate that apparently simple copy-number losses identified by CMA often conceal complex rearrangements, including chromoanasynthesis and ring chromosomes, which become evident only through FISH. Their findings reinforce that accurate interpretation of modern genomic data requires both orthogonal validation and a thorough understanding of chromosome architecture [9]. Large-scale application of high-resolution copy-number assays also provides valuable population reference data. Schwartz and Best analyzed more than 28,000 prenatal microarrays from low-risk pregnancies, establishing baseline frequencies of pathogenic copy-number variants (CNVs), regions of homozygosity, and variants of uncertain significance (VUSs). Their work provides an important empirical benchmark for reproductive medicine and population genomics [10]. Beyond linear DNA sequence, this Special Issue highlights the growing importance of three-dimensional genome organization. Federico et al. review how chromosome territories and higher-order chromatin architecture regulate gene expression, genome stability, and cellular identity, and discuss emerging technologies for three-dimensional genome and epigenome profiling. They illustrate the transformative potential of multi-omics and artificial intelligence in advancing precision diagnostics while emphasizing the indispensable role of cytogenetics and cytogenomics in elucidating genome biology [11]. Extending this concept into clinical practice, Liang et al. use Hi-C sequencing to identify clinically relevant gene fusions and complex structural rearrangements in atypical lymphoma cases that are unresolvable by conventional FISH. Their work demonstrates the diagnostic potential of genome conformation-based technologies as powerful complements to established cytogenetic methods [12]. Advances in structural variant detection are also transforming precision oncology. Chakraborty et al. show that OGM substantially improves risk stratification in chronic lymphocytic leukemia by identifying cryptic translocations, microdeletions, and complex genomic rearrangements beyond the resolution of conventional assays. Integration of OGM with targeted sequencing, IGHV mutational status, and clinical parameters further enhances individualized patient management. Their work provides a blueprint for how comprehensive structural variant profiling is advancing genomic medicine toward integrated, data-driven precision oncology [13]. Finally, Al-Mahrami et al. demonstrate the growing role of artificial intelligence in cytogenomics through ChromoCheck, a support vector machine-based platform that predicts neonatal chromosomal trisomies using multimodal clinical data. This computational model achieves diagnostic performance comparable to conventional karyotyping while providing a scalable decision-support tool for early diagnosis [14].

Edition I of this Special Issue bridges foundational cytogenetic principles with cutting-edge genomic technologies, emphasizing the enduring importance of chromosome biology in genomic medicine. During the preparation of this Editorial, Henry H. Heng published a thought-provoking book proposing that genes and genomes should be regarded as distinct biological entities, offering a conceptual framework for rethinking genetics, evolution, and precision medicine [15]. At the same time, Thomas Liehr launched *ChromoSomics*, a new journal dedicated to chromosome architecture and genome biology across all species [16].

Understanding human disease requires integrating nucleotide-level variation with chromosome-level genome architecture. The outstanding scientific quality of, and enthusiastic response to, Edition I reaffirm the central role of clinical cytogenetics and cytogenomics in modern genomic medicine. Building on this success, I am delighted to announce the launch of Edition II, which will continue to provide a dedicated forum for advances in the study of chromosomal abnormalities associated with developmental disorders, neuropsychiatric diseases, and cancer. Particular emphasis will be placed on emerging genome mapping technologies, long-read sequencing, three- and four-dimensional genome architecture, and the expanding role of artificial intelligence in cytogenetics.

Finally, on behalf of the journal’s editorial team, I extend my sincere gratitude to all authors, reviewers, and colleagues whose expertise and dedication made Edition I of this Special Issue possible. Together, these contributions reinforce the central role of chromosome biology in advancing precision diagnostics, targeted therapies, and the future of genomic medicine.
